# Prescribed medications for patients with amphetamine-type stimulant use disorder seen in rural-serving Pacific Northwest primary care clinics

**DOI:** 10.1186/s13722-025-00593-8

**Published:** 2025-08-13

**Authors:** Megan J. Yerton, Connor J. McCabe, Matthew D. Iles-Shih, Judith I. Tsui, Kevin A. Hallgren

**Affiliations:** 1 University of Washington School of Medicine, Seattle, WA 98195 USA; 2 Department of Psychiatry and Behavioral Sciences, University of Washington, Seattle, WA 98195 USA; 3 Department of General Internal Medicine, University of Washington School of Medicine, Seattle, WA 98104 USA

**Keywords:** Methamphetamine, Pharmacotherapy, Primary care, Rural, Stimulant use disorder

## Abstract

**Background:**

Amphetamine-type stimulant use and overdoses have increased sharply across the US in recent years, largely driven by methamphetamine. Increased access to treatments for amphetamine-type stimulant use disorder (AT-StUD), including in primary care settings, is needed to mitigate these problems, yet effective behavioral treatments are often inaccessible and there are no FDA-approved medications for AT-StUD. In the current study, we characterize how often patients with clinically documented AT-StUD in predominantly rural-serving Pacific Northwest primary care clinics received medications that have been conditionally recommended in practice guidelines for treatment of AT-StUD.

**Methods:**

Electronic health record data from 23 primary care clinics in the Pacific Northwest US were obtained through the Data QUEST network. Adult patients with clinically documented “other stimulant abuse” or “other stimulant dependence” diagnoses typically reflecting AT-StUD between 01/2017 and 12/2021 were included. Prescription records were used to identify orders for bupropion, mirtazapine, topiramate, naltrexone-bupropion combination, methylphenidate, dextroamphetamine, and modafinil. Statistical analyses quantified the percentage of patients with medication orders placed within one year after any documented AT-StUD diagnosis.

**Results:**

Patients (N = 963) were predominantly female (53.3%), White (81.7%), and non-Hispanic (70.5%). In total, 14.3% of patients received orders for a non-stimulant medication conditionally recommended in practice guidelines; 2.7% received orders for a stimulant medication. Consistent with clinical guidelines, medications were more often prescribed when patients had documented co-occurring disorders for which the medications could also be effective.

**Conclusions:**

In this sample of rural-serving primary care clinics, approximately 1 in 7 primary care patients with AT-StUD received orders for medications with preliminary evidence of effectiveness. Efforts are needed to increase access to AT-StUD treatments within primary care. These efforts could include training health professionals to consider judicious use of pharmacotherapy consistent with clinical guidelines, increasing capacity for behavioral health services including contingency management, and continuing research on pharmacologic agents.

**Supplementary Information:**

The online version contains supplementary material available at 10.1186/s13722-025-00593-8.

## Introduction

Amphetamine-type stimulant use (including methamphetamine use) and amphetamine-type stimulant use disorders (AT-StUD) are rising across the United States and increasingly contributing to overdose fatalities [1]. People living in rural areas are disproportionately impacted, reporting nearly twice the likelihood of past-year methamphetamine use (0.95%) compared to those in urban settings (0.52%) [2, 3]. Many people who use amphetamine-type stimulants report a desire for medication-based treatments for AT-StUDs [4]. Although several studies provide preliminary evidence for the effectiveness of medications for AT-StUDs [4–6], there are not yet any US Food and Drug Administration (FDA)-approved medications for the condition. As a result, primary care providers frequently report feeling limited in their ability to help patients with AT-StUD and many providers report using a range of treatment approaches to address AT-StUD, including off-label prescribing (i.e., ordering medications that are FDA approved for conditions other than AT-StUD, with the intention of treating AT-StUD) [7].

In 2023, the American Society of Addiction Medicine (ASAM) and American Academy of Addiction Psychiatry (AAAP) jointly issued their *Clinical Practice Guideline on the Management of Stimulant Use Disorder* [8]. The guideline includes a strong recommendation for offering contingency management (alone or in combination with other psychosocial interventions) to treat AT-StUDs given its strong evidence base [9]. Nonetheless, the guideline also acknowledges that access to contingency management is greatly limited for most people due to implementation barriers (e.g., regulatory, financial, stakeholder buy-in, program resources). Given the limited reach of contingency management, the ASAM and AAAP clinical guidelines also include conditional recommendations for providers to consider the use of medications that have demonstrated preliminary evidence of benefit in clinical trials and that are approved by the FDA for conditions other than AT-StUDs (summarized in Box 1). These medications include bupropion, topiramate, mirtazapine, and combined injectable naltrexone with oral bupropion, with the suggestion to particularly consider these medications when they would also potentially help treat a co-occurring disorder. For example, specific conditional recommendations include offering bupropion if there is a co-occurring tobacco use disorder or depressive disorder, offering mirtazapine if there is a co-occurring depressive disorder, offering topiramate if there is a co-occurring alcohol use disorder, or offering combination injectable naltrexone and oral bupropion if there is a tobacco use disorder, depressive disorder, or alcohol use disorder [8]. The clinical guidelines also include conditional recommendations that physicians can consider prescribing psychostimulants, such as long-acting methylphenidate, particularly for patients with co-occurring attention-deficit/hyperactivity disorder (ADHD). Of note, some states have enacted rules/regulations for prescribing psychostimulants, including restrictions or prohibition of prescribing them to patients with a history of substance use disorders [10]. While no such restrictions impacted prescribing practices in this study, they may well in other regions or contexts.

Prescribing medications for an indication not approved for by the FDA (“off-label” prescribing) is common across medicine [11], including for the treatment of other substance use disorders (e.g., topiramate or gabapentin for alcohol use disorder [12–14]. However, little is known about how often primary care providers prescribe medications to treat AT-StUDs. To better understand these practices, we performed a preliminary, retrospective observational study of patients from predominantly rural-serving primary care clinics with the aim of evaluating how frequently patients with clinically recognized “other stimulant abuse” and “other stimulant dependence”—diagnosis codes that usually reflect methamphetamine use disorder [15]—received orders for medications with preliminary evidence of effectiveness in treating AT-StUDs that were conditionally recommended in practice guidelines [8]. Secondarily, we also examined whether medications were more commonly prescribed in patients who had co-occurring disorders that can also be addressed with the medications.

## Method

### Setting and participants

We obtained data from electronic health records of 23 primary care clinics in the Pacific Northwest US associated with the Data Query Extraction Standardization and Translation (Data QUEST) network—a network of practices that receive technical support for harmonizing electronic health record data to advance translational research [16, 17]. The primary care clinics participating in the study were community health clinics that served patients with limited income, as well as rural hospital-associated clinics that mostly served patients in rural areas and small cities. The sample included all adult patients with International Classification of Disease (ICD)−9 or ICD-10 coded diagnoses of “other stimulant abuse” or “other stimulant dependence” between January 2017 and December 2021 (ICD codes included in the online supplement). There is no ICD code specifically for methamphetamine use disorder; however, a previous study showed that 79–92% of patients with ICD codes reflecting other stimulant use disorder had methamphetamine use [15].

### Measures

Descriptive measures included demographics, records of encounters with behavioral health specialists (e.g., psychologist, social worker, mental health counselor), ICD codes for co-occurring mental health and substance use disorders specifically mentioned in the ASAM and AAAP clinical guidelines for which providers were recommended to particularly consider pharmacotherapy (depressive disorder, ADHD, tobacco use disorder, alcohol use disorder), and ICD codes for any mental health condition and for any substance use disorder (excluding other stimulant use disorders and tobacco use disorders).

The primary outcome measures were electronic health record orders for medications with preliminary evidence of effectiveness for AT-StUDs [4–6] that were conditionally recommended in the 2023 ASAM and AAAP clinical guidelines [8]. We identified patients with orders for these medications 0 to 365 days after any ICD coded “other stimulant disorder” diagnosis made within a participating primary care clinic. Patients who had multiple instances of ICD coded other stimulant abuse or dependence were flagged as having received a medication order if one was made 0 to 365 days after any of the documented other stimulant abuse or dependence diagnoses. Patients who had an order for naltrexone within 30 days of an order for oral bupropion were considered to have received naltrexone-bupropion combination; for patients receiving this combination, we further characterized whether orders were for injectable naltrexone (recommended by the ASAM and AAAP clinical guidelines) or oral naltrexone (not recommended). Similarly, we identified patients who received methylphenidate in any form, then specifically examined whether they received orders for a long-acting formula (recommended by the ASAM the AAAP clinical guidelines). Although the ASAM and AAAP clinical guidelines did not conditionally recommend other psychostimulants, we examined receipt of orders for two other psychostimulants that have demonstrated preliminary evidence of potential effectiveness in clinical trials, namely modafinil and dextroamphetamine [4–6].

### Analysis plan

Descriptive statistics were calculated to characterize the percentage of patients with clinically documented other stimulant use disorders who received medications and/or had a visit with a behavioral health specialist within 0–365 days after the clinically documented other stimulant use disorder diagnosis.

Inferential analyses also examined whether patients were more likely to receive medications when they also had a clinically documented co-occurring mental health or substance use disorder for which the ASAM and AAAP clinical guidelines offered conditional recommendations for additional consideration of medications. Specifically, we used Fisher exact tests—nonparametric statistical tests suitable for testing differences in categorical variables even if expected cell sizes are small [18] to examine whether patients with documented tobacco use disorders were more likely to receive orders for bupropion or naltrexone-bupropion combination, whether patients with documented depressive disorders were more likely to receive orders for bupropion, mirtazapine, or naltrexone-bupropion combination, whether patients with documented alcohol use disorders were more likely to receive topiramate or naltrexone-bupropion combination, and whether patients with documented ADHD were more likely to receive methylphenidate. We also examined whether patients with documented ADHD were more likely to receive modafinil or dextroamphetamine.

## Results

### Descriptive statistics

There were 963 adult primary care patients who met the study inclusion criteria (Table 1). Approximately half (49.5%) were between the ages of 30 and 49. A slight majority were female (53.3%); most were White (81.7%) and non-Hispanic (70.5%). Over half of the sample (57.7%) had a co-occurring ICD coded mental health disorder and over one-third of the sample (37.6%) had an ICD coded non-tobacco substance use disorder in addition to other stimulant abuse or dependence (Table 1).

**Table 1:** Demographics, co-occurring conditions, and encounters with behavioral health specialists among primary care patients in rural-serving clinics with clinically documented other stimulant abuse or dependence (N = 963)

	All Patients (N = 963)	Received Medication (N = 159)	Did Not Receive Medication (N = 804)
	N (%)	N (%)	N (%)
Age			
18–29	226 (23.5)	36 (22.9)	190 (24.0)
30–49	477 (49.5)	87 (55.4)	390 (49.3)
≥ 50	245 (25.4)	34 (21.7)	211 (26.7)
Unknown	15 (1.6)	2 (1.3)	13 (1.6)
Sex			
Female	513 (53.3)	90 (56.7)	423 (42.6)
Male	450 (46.7)	69 (43.4)	381 (47.4)
Race			
American Indian or Alaska Native	26 (2.7)	2 (1.3)	24 (3.0)
Asian	3 (0.3)	0 (0.0)	3 (0.4)
Black or African American	14 (1.5)	4 (2.5)	10 (1.2)
Native Hawaiian or Other Pacific Islander	5 (0.5)	0 (0.0)	5 (0.6)
Unknown	128 (13.3)	10 (6.3)	118 (14.7)
White	787 (81.7)	143 (89.9)	644 (80.1)
Ethnicity			
Hispanic or Latino	79 (8.2)	5 (3.1)	74 (9.2)
Not Hispanic or Latino	679 (70.5)	117 (73.6)	562 (69.9)
Unknown/Not Provided	205 (21.3)	37 (23.3)	168 (20.9)
Co-occurring conditions			
Any co-occurring mental health disorder	556 (57.7)	128 (80.5)	428 (53.2)
Any co-occurring substance use disorder (excluding methamphetamine and tobacco use disorders)	362 (37.6)	73 (45.9)	289 (35.9)
Tobacco use disorder	280 (29.1)	52 (32.7)	228 (28.4)
Depressive disorder	327 (34.0)	88 (55.3)	239 (29.7)
Alcohol use disorder	88 (9.1)	24 (15.1)	64 (8.0)
Attention-deficit/hyperactivity disorder (ADHD)	52 (5.4)	24 (15.1)	28 (3.5)
Encounters with behavioral health specialists			
Yes	472 (51.0)	93 (58.5)	379 (47.1)
No	491 (49.0)	66 (41.5)	425 (52.9)

With respect to conditions for which additional consideration of pharmacotherapy is conditionally recommended: 34.0% of the sample had ICD coded depressive disorders, 29.1% had ICD coded tobacco use disorders, 9.1% had ICD coded alcohol use disorders, and 5.4% had ICD coded ADHD. Nearly half of all patients in the sample (49%, n = 472) had one or more encounters with a behavioral health specialist within the year after ICD coded other stimulant abuse or dependence.

Of the total sample, 16.5% of patients received one or more of the medications investigated in this study. Those with co-occurring conditions were more likely to receive an order for one of these medications (Table 1). Of those who received any medication, 80.5% had any co-occurring mental health disorder compared to 53.2% of those who did not receive medication. Of those who received medication, 45.9% had any co-occurring substance use disorder compared to 35.9% of those who did not receive medication.

### Non-stimulant medication orders

14.3% of patients (n = 138 of 963) received orders for any of the non-stimulant medications examined in this study 0–365 days after an EHR-documented other stimulant abuse or dependence diagnosis. The percentages of patients receiving non-stimulant medications are presented in Table 2 for the full sample and for subgroups defined by co-occurring disorders. Bupropion orders were given to 8% of patients (n = 77) and were more commonly ordered for patients with ICD coded depressive disorders than for patients without (14.4% vs. 4.7%, p < 0.001). However, the percentage of patients receiving bupropion orders did not significantly differ between patients with and without ICD coded tobacco use disorders (7.5% vs. 8.2%, p = 0.79). Mirtazapine orders were given to 3.5% of patients (n = 34), with orders being significantly more frequent for patients with ICD coded depressive disorders compared to patients without (5.8% vs. 2.4%, p = 0.009). Topiramate orders were given to 1.9% of patients (n = 18), with orders being significantly more frequent for patients with ICD coded alcohol use disorders compared to patients without (5.7% vs. 1.5%, p = 0.02). Naltrexone-bupropion combination orders were given to 0.4% of patients (n = 4), three of whom received orders for injectable naltrexone and one of whom received orders for oral naltrexone. Receipt of naltrexone-bupropion combination orders was more common in patients with ICD coded alcohol use disorders compared to patients without (2.3% vs. 0.2%, p = 0.04), but did not differ for patients with versus without ICD coded tobacco use disorders (0.7% vs. 0.3%, p = 0.58) or for patients with versus without ICD coded depressive disorders (0.9% vs. 0.2%, p = 0.12).

**Table 2:** Receipt of medication orders among primary care patients with clinically documented other stimulant abuse or dependence (N = 963), for full sample and based on co-occurring disorders

	Receiving one or more of the recommended medications			Any recommended non-stimulant			Bupropion			Mirtazapine			Topiramate			Naltrexone-Bupropion combination			Any recommended stimulant				Methyl-phenidate			Modafinil or Dextroam-phetamine		
	N	(%)		N	(%)		N	(%)		N	(%)		N	(%)		N	(%)		N	(%)			N	(%)		N	(%)	
Full Sample (n = 963)	159	(16.5)		138	(14.3)		77	(8.0)		34	(3.5)		18	(1.9)		4	(0.4)		26	(2.7)			4	(0.4)		23	(2.4)	
Documented tobacco use disorder																												
Yes (n = 280)	52	(18.6)		**47**	(16.8)		**21**	**(7.5)**		14	(5.0)		9	(3.2)		**2**	**(0.7)**		9	(3.2)			0	(0.0)		9	(3.2)	
No (n = 683)	107	(15.7)		**91**	(13.3)		**56**	**(8.2)**		20	(2.9)		9	(1.3)		**2**	**(0.3)**		17	(2.5)			4	(0.6)		14	(2.0)	
Documented depressive disorder																												
Yes (n = 327)	**88**	**(26.9)**	***	**78**	**(23.9)**	***	**47**	**(14.4)**	***	**19**	**(5.8)**	**	9	(2.8)		**3**	**(0.9)**		**14**	**(4.3)**	*		1	(0.3)		13	(4.0)	*
No (n = 636)	**71**	**(11.2)**	***	**60**	**(9.4)**	***	**30**	**(4.7)**	***	**15**	**(2.4)**	**	9	(1.4)		**1**	**(0.2)**		**12**	**(1.9)**	*		3	(0.5)		10	(1.6)	*
Documented alcohol use disorder																												
Yes (n = 88)	**24**	**(27.3)**	**	**22**	**(25.0)**	**	8	(9.1)		6	(6.8)		**5**	**(5.7)**	*	**2**	**(2.3)**	*	2	(2.3)			0	(0.0)		2	(2.3)	
No (n = 875)	**135**	**(15.4)**	**	**116**	**(13.3)**	**	69	(7.9)		28	(3.2)		**13**	**(1.5)**	*	**2**	**(0.2)**	*	24	(2.7)			4	(0.5)		21	(2.4)	
Documented attention-deficit/hyperactivity disorder																												
Yes (n = 52)	**24**	**(46.2)**	***	12	(23.1)		6	(11.5)		1	(1.9)		2	(3.8)		0	(0.0)		**14**	**(26.9)**	***		**2**	**(3.8)**	*	**13**	**(25.0)**	***
No (n = 911)	**135**	**(14.8)**	***	126	(13.8)		71	(7.8)		33	(3.6)		16	(1.8)		4	(0.4)		**12**	**(1.3)**	***		**2**	**(0.2)**	*	**10**	**(1.1)**	***

Values in bold font are used to indicate when a medication is conditionally recommended within the ASAM and AAAP clinical guidelines when a specific co-occurring mental health or substance use disorder is also present. Asterisks indicate significant differences in the incidence of treatments for patients with the co-occurring disorder compared to patients without the co-occurring disorder. *p <.05, **p <.01, ***p <.001

### Stimulant medication orders

2.7% of patients (n = 26 of 963) received orders for any of the stimulant medications examined in this study within 0–365 days after an ICD coded other stimulant abuse or dependence diagnosis. Results for the full sample and for subgroups defined by co-occurring disorders are presented in Table 2. Methylphenidate orders were given to 0.4% of patients (n = 4), all of whom received orders for the long-acting formulation. Receipt of a methylphenidate order was more common in patients with ICD coded ADHD compared to patients without (3.8% vs. 0.2%, p = 0.02). The other two psychostimulants (modafinil or dextroamphetamine) were ordered for 2.4% of patients (n = 23), with orders being significantly more frequent among patients with documented ADHD than for patients without (25.0% vs. 1.1%, p < 0.001).

## Discussion

Findings from this study suggest that up to 1 in 7 patients (14.3%) with clinically recognized other stimulant abuse or dependence in primarily rural-serving Pacific Northwest primary care clinics received an order for a non-stimulant medication that has demonstrated preliminary evidence of effectiveness for treating AT-StUDs (particularly methamphetamine use disorder) [4–6, 19] and that has been conditionally recommended by clinical guidelines for the treatment of AT-StUDs [8]. The study also found that 1 in 37 (2.7%) primary care patients with documented other stimulant abuse or dependence received an order for a stimulant medication. To our awareness, this is the first study to examine the frequency of prescribing medications used to treat AT-StUDs by rural primary care providers. A previous chart review study of 71 patients in a large, urban academic medical center in Massachusetts found that 24% of patients received pharmacotherapy with an explicit indication for harm reduction or AT-StUD treatment and that 49% received a stimulant medication (typically with an unclear indication) [20]. Exact reasons for lower rates of prescribing in the current study are unknown but may reflect regional differences in stimulant prescribing [21], differences in patient populations (e.g., higher rates of depression, tobacco use disorder, and opioid use disorder in the Massachusetts sample), and differences related to rurality and the type of health systems examined (e.g., large, urban academic medical center with specialty addiction services in the Massachusetts sample vs. rural-serving primary care clinics with fewer specialty services in the current sample). Another study using national registry data from Sweden with 13,965 individuals with first-time amphetamine or methamphetamine use disorder found higher rates of some medications (e.g., mirtazapine [19.7%], methylphenidate [21.8%]) and lower rates for other medications (e.g., bupropion [6.9%], topiramate [0.8%]) – potential reasons for these differences could include the Swedish study sample including records from inpatient and specialty outpatient settings (vs. primary care) and different policies and practices around stimulant use disorder treatment and stimulant prescribing in Sweden compared to the United States [22].

This study’s findings further indicate that primary care patients with documented depressive disorders, alcohol use disorders, or ADHD had a significantly higher probability of receiving medications with preliminary evidence of effectiveness for AT-StUDs, which is consistent with ASAM and AAAP’s conditional recommendations for providers to give additional consideration to medications when the medication could also be used to treat a co-occurring condition. An exception, however, is that patients with documented tobacco use disorders were not more likely to receive bupropion and naltrexone-bupropion combination compared to patients without documented tobacco use disorders. Reasons for this are unknown, but one possibility is that it reflects a national trend of decreasing use of medications for smoking cessation that has been observed for the general US adult population [23]. We are not aware of studies documenting the extent to which medications for smoking cessation are prescribed to people with AT-StUDs, but given the high rates of tobacco use disorder in this sample (29.1%) and high rates of tobacco use among US adults with substance use disorders [24], additional efforts are warranted to increase access to smoking cessation resources for primary care patients with AT-StUDs and tobacco use disorders, particularly bupropion which preliminary evidence suggests may help some patients stop or reduce their use of methamphetamine [6, 19].

With no FDA-approved medications for AT-StUDs, additional efforts are needed to support primary care providers in treating AT-StUDs. These efforts could include training providers to consider judicious use of pharmacotherapy in accordance with current practice guidelines [8] and obtaining consultation from addiction medicine specialists. Efforts could also include developing approaches to increase the capacity for contingency management within primary care settings, potentially with the use of mobile health technology to reduce potential primary care staff burden and to mitigate barriers that disproportionately limit access to care for people living in rural areas (e.g., transportation, childcare) [25–27]. Efforts to support primary care providers in addressing AT-StUD are particularly warranted in rural communities, where substance use disorder treatment options outside of primary care are more limited [28] and where substance use disorder treatment programs have less capacity to offer pharmacotherapy, wraparound services, and specialized treatment options [29].

### Limitations

This study has important limitations. The medication orders data did not include information about whether medications were intended to treat AT-StUDs and/or any other co-occurring conditions. Thus, we cannot know whether providers ordered medications with the intention of treating an AT-StUD and/or another co-occurring condition. Since there is no ICD-9 or ICD-10 code to indicate the type of stimulant being used (e.g., methamphetamine versus another amphetamine-type stimulant), the sample likely included patients with different types of AT-StUDs, although it is likely that most used methamphetamine [15]. In light of these limitations, findings from this study should be viewed as providing preliminary insights about orders for medications that *could have potentially been intended* to treat AT-StUDs.

The study sample was predominantly white and non-Hispanic, which reflects the demographic makeup of the rural Pacific Northwest; however, findings may not generalize to more diverse rural populations, including those in other regions. Prescribing practices can vary by geographical region for some of the medications studied here (e.g., psychostimulants) [21]. The study data did not include information about treatment services that could have been received outside of the primary care settings that were included (e.g., specialty substance use disorder treatment). The low incidence of some medication orders likely limited statistical power for some analyses—particularly for analyses testing whether naltrexone-bupropion combination and methylphenidate were more commonly prescribed in patients with co-occurring disorders. Additionally, this analysis did not account for multiple hypothesis testing, increasing the risk of type I error.

The study also has noteworthy strengths. The sample included a large number of patients with clinically recognized AT-StUDs. The data were obtained from a practice-based research network that included 23 primary care clinics in the Pacific Northwest of the US—a region where the prevalence of methamphetamine use disorder has been particularly high for many years [1]. The study also focused on rural-serving primary care clinics, where resources for addressing AT-StUDs are particularly limited.

## Conclusions

Our study using data from rural-serving primary care clinics found that roughly 1 in 7 primary care patients with clinically recognized AT-StUDs received an order for a medication that could have potentially been used to treat their AT-StUD. Future efforts should focus on increasing access to AT-StUD treatments within primary care. Such efforts may include training health professionals to consider using pharmacotherapy judiciously in a manner consistent with clinical guidelines, increasing capacity for behavioral health services including contingency management, and continuing research to develop and test medications for AT-StUDs.

Box 1 Summary of conditional recommendations for pharmacotherapy-based treatment of amphetamine-type stimulant use disorders (AT-StUDs) by the American Society of Addiction Medicine (ASAM) and American Academy of Addiction Psychiatry (AAAP). See the *Clinical Practice Guideline on the Management of Stimulant Use Disorder* for full recommendations [8].

- *Bupropion* Low certainty, conditional recommendation for patients with amphetamine-type stimulant use disorder with low- to moderate-frequency of methamphetamine use (< 18 days per month). Additional consideration for patients with co-occurring tobacco use disorder (low certainty, conditional recommendation) and/or co-occurring depressive disorders (low certainty, conditional recommendation).
- *Naltrexone-bupropion combination* Moderate certainty, conditional recommendation for patients with amphetamine-type stimulant use disorder. Additional consideration for patients with co-occurring alcohol use disorder (moderate certainty, conditional recommendation), tobacco use disorder (moderate certainty, conditional recommendation), and/or depression (moderate certainty, conditional recommendation).
- *Topiramate* Low certainty, conditional recommendation for patients with amphetamine-type stimulant use disorder. Additional consideration for patients with co-occurring alcohol use disorder (low certainty, conditional recommendation).
- *Mirtazapine* Low certainty, conditional recommendation for patients with amphetamine-type stimulant use disorder. Additional consideration for patients with co-occurring depressive disorders (low certainty, conditional recommendation).
- *Methylphenidate (long-acting formulation)* Low certainty, conditional recommendation for patients with amphetamine-type stimulant use disorder. Additional consideration for patients with moderate or higher frequency of amphetamine-type stimulant use disorder at treatment start (≥ 10 days per month, low certainty conditional recommendation). Additional consideration for patients with co-occurring ADHD (low certainty, conditional recommendation). Recommendations related to prescription of psychostimulant medications to treat stimulant use disorder are only applicable to physician specialists who are board certified in addiction medicine or addiction psychiatry and who have “commensurate training, competencies, and capacity for close patient monitoring” (clinical consensus, strong recommendation, p. 60). Physicians who prescribe long-acting methylphenidate should maintain a level of monitoring commensurate with the risk profile for the medication and the patient (e.g., pill counts, substance testing, frequent clinical contact, frequent prescription drug monitoring program checks; clinical consensus, strong recommendation).

## Supplementary Information

Additional file 1

## Data Availability

Research data reported in this manuscript is subject to a data use agreement with the Data Query Extraction Standardization and Translation (Data QUEST) network. The authors are prohibited from sharing the data received under this agreement with other parties. Requests for data from Data QUEST may be placed at the following website: https://familymedicine.uw.edu/dataquest/get-started/

## Abbreviations

ADHD: Attention-deficit/hyperactivity disorder
ASAM: American Society of Addiction Medicine
AAAP: American Academy of Addiction Psychiatry
FDA: Food and Drug Administration
ICD: International Classification of Disease
AT-StUD: Amphetamine-type stimulant use disorder
US: United States
