# Supplementary material for: Prescribed medications for patients with amphetamine-type stimulant use disorder seen in rural-serving Pacific Northwest primary care clinics
Source: Addict Sci Clin Pract. 2025 Aug 13;20:67. doi: 10.1186/s13722-025-00593-8 (PMC12344905; doi:10.1186/s13722-025-00593-8)
Supplement: Supplementary file 1 — Additional file 1 [file 13722_2025_593_MOESM1_ESM.docx]

| **Description** | **Code** | **ICD version** |
| --- | --- | --- |
| Other stimulant abuse, uncomplicated | F15.10 | ICD10 |
| Other stimulant abuse with intoxication, uncomplicated | F15.120 | ICD10 |
| Other stimulant abuse with intoxication delirium | F15.121 | ICD10 |
| Other stimulant abuse with intoxication with perceptual disturbance | F15.122 | ICD10 |
| Other stimulant abuse with intoxication, unspecified | F15.129 | ICD10 |
| Other stimulant abuse with stimulant-induced mood disorder | F15.14 | ICD10 |
| Other stimulant abuse with stimulant-induced psychotic disorder with delusions | F15.150 | ICD10 |
| Other stimulant abuse with stimulant-induced psychotic disorder with hallucinations | F15.151 | ICD10 |
| Other stimulant abuse with stimulant-induced psychotic disorder, unspecified | F15.159 | ICD10 |
| Other stimulant abuse with stimulant-induced anxiety disorder | F15.180 | ICD10 |
| Other stimulant abuse with stimulant-induced sexual dysfunction | F15.181 | ICD10 |
| Other stimulant abuse with stimulant-induced sleep disorder | F15.182 | ICD10 |
| Other stimulant abuse with other stimulant-induced disorder | F15.188 | ICD10 |
| Other stimulant abuse with unspecified stimulant-induced disorder | F15.19 | ICD10 |
| Other stimulant dependence, uncomplicated | F15.20 | ICD10 |
| Other stimulant dependence, in remission | F15.21 | ICD10 |
| Other stimulant dependence with intoxication, uncomplicated | F15.220 | ICD10 |
| Other stimulant dependence with intoxication delirium | F15.221 | ICD10 |
| Other stimulant dependence with intoxication with perceptual disturbance | F15.222 | ICD10 |
| Other stimulant dependence with intoxication, unspecified | F15.229 | ICD10 |
| Other stimulant dependence with withdrawal | F15.23 | ICD10 |
| Other stimulant dependence with stimulant-induced mood disorder | F15.24 | ICD10 |
| Other stimulant dependence with stimulant-induced psychotic disorder with delusions | F15.250 | ICD10 |
| Other stimulant dependence with stimulant-induced psychotic disorder with hallucinations | F15.251 | ICD10 |
| Other stimulant dependence with stimulant-induced psychotic disorder, unspecified | F15.259 | ICD10 |
| Other stimulant dependence with stimulant-induced anxiety disorder | F15.280 | ICD10 |
| Other stimulant dependence with stimulant-induced sexual dysfunction | F15.281 | ICD10 |
| Other stimulant dependence with stimulant-induced sleep disorder | F15.282 | ICD10 |
| Other stimulant dependence with other stimulant-induced disorder | F15.288 | ICD10 |
| Other stimulant dependence with unspecified stimulant-induced disorder | F15.29 | ICD10 |
| Other stimulant use, unspecified with other stimulant-induced disorder | F15.988 | ICD10 |
| Amphetamine and other psychostimulant dependence | 304.4 | ICD9 |
| Amphetamine and other psychostimulant dependence, unspecified | 304.40 | ICD9 |
| Amphetamine and other psychostimulant dependence, continuous | 304.41 | ICD9 |
| Amphetamine and other psychostimulant dependence, episodic | 304.42 | ICD9 |
| Amphetamine and other psychostimulant dependence, in remission | 304.43 | ICD9 |
| Amphetamine or related acting sympathomimetic abuse | 305.7 | ICD9 |
| Amphetamine or related acting sympathomimetic abuse, unspecified | 305.70 | ICD9 |
| Amphetamine or related acting sympathomimetic abuse, continuous | 305.71 | ICD9 |
| Amphetamine or related acting sympathomimetic abuse, episodic | 305.72 | ICD9 |
| Amphetamine or related acting sympathomimetic abuse, in remission | 305.73 | ICD9 |
